# Compressive and tensile properties of polyurethane foam mimicking trabecular tissue in artificial femoral head bones

**DOI:** 10.3389/fbioe.2024.1482165

**Published:** 2025-01-17

**Authors:** Krzysztof Zerdzicki, Aleksander Znaczko, Aleksandra Kondrusik, Wiktoria Korbut

**Affiliations:** Gdansk University of Technology, Faculty of Civil and Environmental Engineering, Department of Structural Mechanics, Gdansk, Poland

**Keywords:** femurs, artificial bone, polyurethane foam, sawbones, synbone, trabecular tissue, mechanical testing

## Abstract

**Introduction:**

Most orthopedic implants for fracture fixation and joint replacement are commonly tested on artificial bones. Polyurethane foam is one of the most frequently used synthetic bone materials for mimicking human trabecular tissue. The study aimed to examine the compressive and tensile behavior of polyurethane foams mimicking trabecular tissue in artificial human femurs and assess their potential to replicate the osteoporotic type of human bone tissue.

**Materials and methods:**

Two types of SYNBONE femur models, one of normal density (model 2350) and one of lower density (model LD2350), and three types of Sawbones femur models (models 1130-21-8, 1130-21-3, and 1130-192) were investigated. Polyurethane foams were extracted as slices cut in the coronal plane from the femoral head. Cuboid samples were cut in three directions and uniaxial tested to identify compressive and tensile properties, including elastic modulus, Poisson’s ratio, yield limit, and ultimate strength.

**Results:**

The ANOVA test revealed that only SYNBONE LD2350 trabecular tissue exhibits anisotropy properties (*p* < 0.001). In most cases, the tensile properties were greater than compressive ones (t-test, *p* < 0.001).

**Conclusion:**

The obtained results are within the ranges suggested by other authors for mimicking osteoporotic human tissue. The presented data broadens the range of data on mechanical properties provided by the producers. These data can serve as a reference for research using composite femurs made of these particular polyurethane foams for conducting biomechanical studies and validating accompanying numerical simulations.

## 1 Introduction

Most orthopedic implants for fracture fixation and joint replacement are commonly tested on artificial bones. Synthetic bones are easily available, cheap, infection-free, and guarantee reproducibility of shape and mechanical properties compared to natural bones (Shim et al., 2012). However, synthetic bones are more homogenous and less anisotropic than natural bones (Calvert et al., 2010). Polyurethane foam is one of the most frequently used synthetic bone materials for mimicking human trabecular tissue. It can be obtained in various densities and forms of inner cells, giving solid, cellular, and open foam types (Marter et al., 2019) (Muhayudin et al., 2020). The mechanical behavior of cancellous bone and polyurethane foams exhibit similar responses that can be seen in three characteristic regimes in the typical compressive stress–strain curve (Shim et al., 2012) (Calvert et al., 2010) (Szivek et al., 1993; Szivek et al., 1995). First, elastic behavior is observed, followed by a plateau region when the cells collapse, which finally results in material hardening due to the densification effect related to the closing of the cells. Over the years, many modifications and improvements have been made in the production of synthetic bones, such as the application of epoxy resin or short fiberglass. Many tests have been conducted on the whole composite femur to meet the natural bone behavior under various loading scenarios (Heiner and Brown, 2001; Heiner, 2008). An additional challenge is to simulate the behavior of the osteoporotic bone experimentally and numerically, as most implants for fracture fixation and joint replacement are anchored in the diseased bones of older people.

The PU foam composite bones are produced by different vendors, including SYNBONE, Sawbones, General Plastics (GP), and Bonesim (Brown et al., 2019). The relatively cheap polyurethane bone analogs are generally used in surgical training as they reflect true bone geometry and overall rigidity and provide surgeons with the feeling of humanlike bone regarding drilling, sawing, taping, and plating. However, increasing numbers of biomechanical studies are being conducted on these bones. SYNBONE bones were used for the analysis of different implants and cerclage augmentation for intertrochanteric fractures (Ceynowa et al., 2020a) (Ceynowa et al., 2021a), cement-bone bonds (Id et al., 2021), evaluation of drill hole influence on bone strength (Ceynowa et al., 2020b), and ballistic tests (Appleby-Thomas et al., 2016), (Riva et al., 2019) (foam blocks). Third- and fourth-generation Sawbone composite bones (short glass fiber reinforced epoxy resin + PU foam) are frequently used in biomechanical analysis, as they have been positively validated with human bones many times [(Zdero et al., 2023), (Lamb et al., 2022), (Gluek et al., 2020), (Aziz et al., 2014), (Nicayenzi et al., 2012), (Dunlap et al., 2008), (Domann et al., 2011)] and were systematically developed over the years. SYNBONE and Sawbones calcaneal bone analogs were compared with embalmed and fresh frozen human bones under cyclic loading to observe differences in their mechanical behavior (Zech et al., 2006).

At the same time, increasing numbers of biomechanical simulations of femur–implant constructs are performed by numerical calculations and advanced computing systems. These numerical models should be experimentally validated on real human bones [(Enns-Bray et al., 2016) (Op Den Buijs and Dragomir-Daescu, 2011) (Trabelsi et al., 2011) (Yosibash et al., 2007)] or at least on artificial bone analogs during laboratory tests to prove their correctness. It is of key importance to know the properties of the artificial bones that are used for laboratory tests and accompanying validation. Such validated model recalculated with true human bone properties should give results similar to the behavior of real human bone. That should be a common practice to obtain validated, reliable results and draw forensic conclusions of clinical importance.

The mechanical properties of synthetic bones are usually evaluated based on experiments conducted on specimens cut out of large blocks of foam supplied by the manufacturers with known foam densities (Brown et al., 2019) (Marter et al., 2019) (Calvert et al., 2010) (Patel et al., 2008). However, the properties of the foams in the final ready-to-use bone analog product may differ. More data on cancellous bone analogs are required, as the producers give only partial details or focus on the cortical bone properties (Zdero et al., 2023). There is a lack of information on the properties of cancellous bone analogs in the whole bone constructs.

The main aim of the current study was to investigate the mechanical properties of the inner part of the composite bones resembling trabecular tissue in the femoral head for selected bone analogs of two top producers offering osteoporotic types of composite femurs. The study covered behavior under static loading of both compressive and tensile characters. The additional aims were to evaluate the isotropic character of polyurethane foams mimicking the trabecular tissue in the femoral head bone analogs and to compare the obtained results with the osteoporotic properties of the natural human femur bone.

## 2 Materials and methods

### 2.1 Sample preparation

**FIGURE 1:**
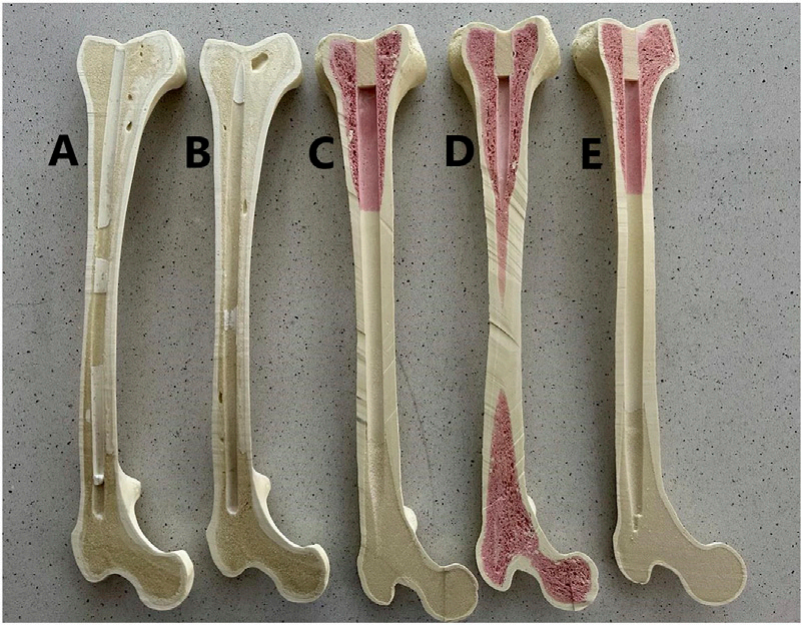
Cross sections along the coronal plane of the tested synthetic femurs: **(A)** SYNBONE LD2350, **(B)** SYNBONE 2350, **(C)** Sawbones 1130-21-3, **(D)** Sawbones 1130-192, and **(E)** Sawbones 1130-21-8.

Selected synthetic femurs from SYNBONE Inc. (Davos, Switzerland) and Sawbones (Sawbones Inc., Vashon, WA, USA) were analyzed. The key rule for selecting the femurs was their possibility of being used as analogs for osteoporotic bone. The left femur was always taken for experiments. The manufacturers’ specifications of the tested femurs are collected in Table 1., and the cross-sections along the coronal plane of the tested femurs are presented in Figure 1. All specimens were stored and tested at room temperature and normal humidity conditions.

**TABLE 1:** Specification of the investigated synthetic femurs, sawbones.com, synbone.com.

Bone model	Geometry	Materials	Application
SYNBONE 2350	Length: 460 mm. Condylar width: 85 mm. Neck angle: 120°. Anteversion: 18°. Head diameter: 49 mm. Canal diameter: 10 mm	Cortical/soft cancellous bone	Screwing and plating, nailing
SYNBONE LD2350	Length: 460 mm. Condylar width: 85 mm. Neck angle: 120°. Anteversion: 18°. Head diameter: 49 mm. Canal diameter: 10 mm	Cortical low-density/soft cancellous bone	Screwing and plating, nailing
Sawbones 1130-21-8		Solid rigid foam cortical shell Femur with 7 PCF (0.11 g/cm^3^) cancellous inner material at the proximal end. Distal end with standard cancellous material	
Sawbones 1130-21-3	Canal diameter of 16 mm and an overall length of 47 cm	Solid rigid foam cortical shell Large left with 3 PCF (0.05 g/cm^3^) light-density foam The femur includes 3 PCF cancellous material in the proximal end and standard cancellous material in the distal end	Works well for broaching and insertion of implants. Ideal for short stems
Sawbones 1130-192	Canal diameter of 10 mm and an overall length of 47 cm	Solid rigid foam cortical shell The femur includes cancellous inner material	

**FIGURE 2:**
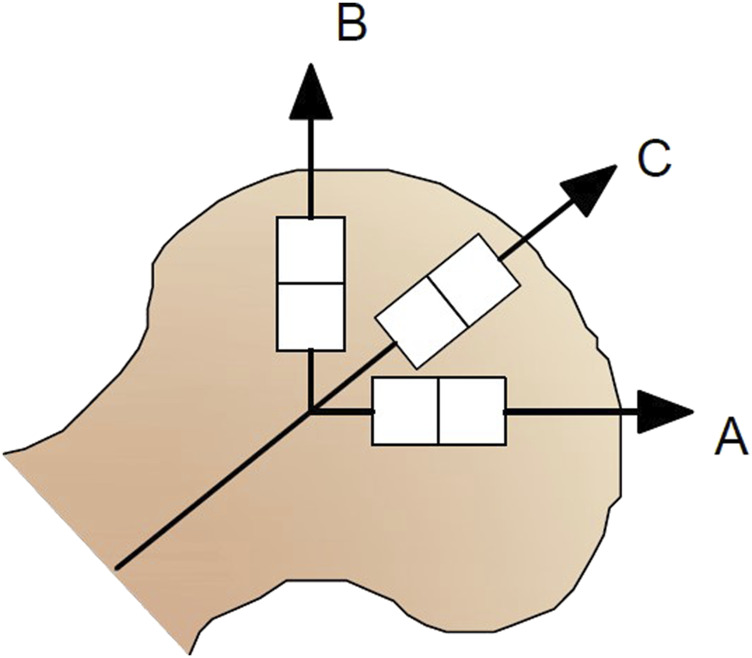
Specimen cutting and marking the directions analyzed in the study.

Two specimen-preparation methodologies were introduced. PU foams were retrieved from the inner part of the femoral head. First, the femoral head was placed in a special holder with the band saw and cut into 4-mm-thick slices in the coronal plane without cutting off the slices out of the femoral shaft to keep them in the original position in the head. Each artificial femoral head was mounted in the holder in the same way. Then, the slices were cut by a band saw into strips of 10 mm width and 4 mm thickness in three directions (Figure 2): along the horizontal plane (A), along the vertical plane (B), and along the femoral neck slope (C). For the compression tests, the specimens’ planes parallel to the machine clamps were always cut with a band saw to guarantee their parallelism. Additionally, the compressed surfaces of specimens were gently polished with fine-grained sandpaper. Planes perpendicular to testing machine clamps were cut by a carpenter knife and also polished with fine-grained sandpaper to create cuboid-shaped specimens. For the tensile tests, both sides of cuboid specimens parallel to the loading direction were cut by the band saw when the femoral head bone was held in the special handle. Before size and weight measurement, all samples were gently cleaned of dust using a vacuum cleaner.

For the compression tests, the final samples were approximately 10 mm × 10 mm × 4 mm, according to ISO 604 standard requirements (EN ISO 604, 2002). For tensile tests, the specimens had final dimensions of approximately 10 mm × 40–50 mm × 4 mm, which guaranteed that the cross section of the compression and tensile samples was the same. Then, the samples were measured three times in each direction (height, width, length) by an electronic caliper (LIMIT, Sweden, accuracy 0.02 mm) and weight three times (LIMIT, Sweden, Iem-7, accuracy 0.01 g). The apparent density was calculated as the mass/volume relationship, where volume was obtained based on the measurements and upon the assumption that samples were ideally cuboid-shaped.

### 2.2 Testing procedure

**FIGURE 3:**
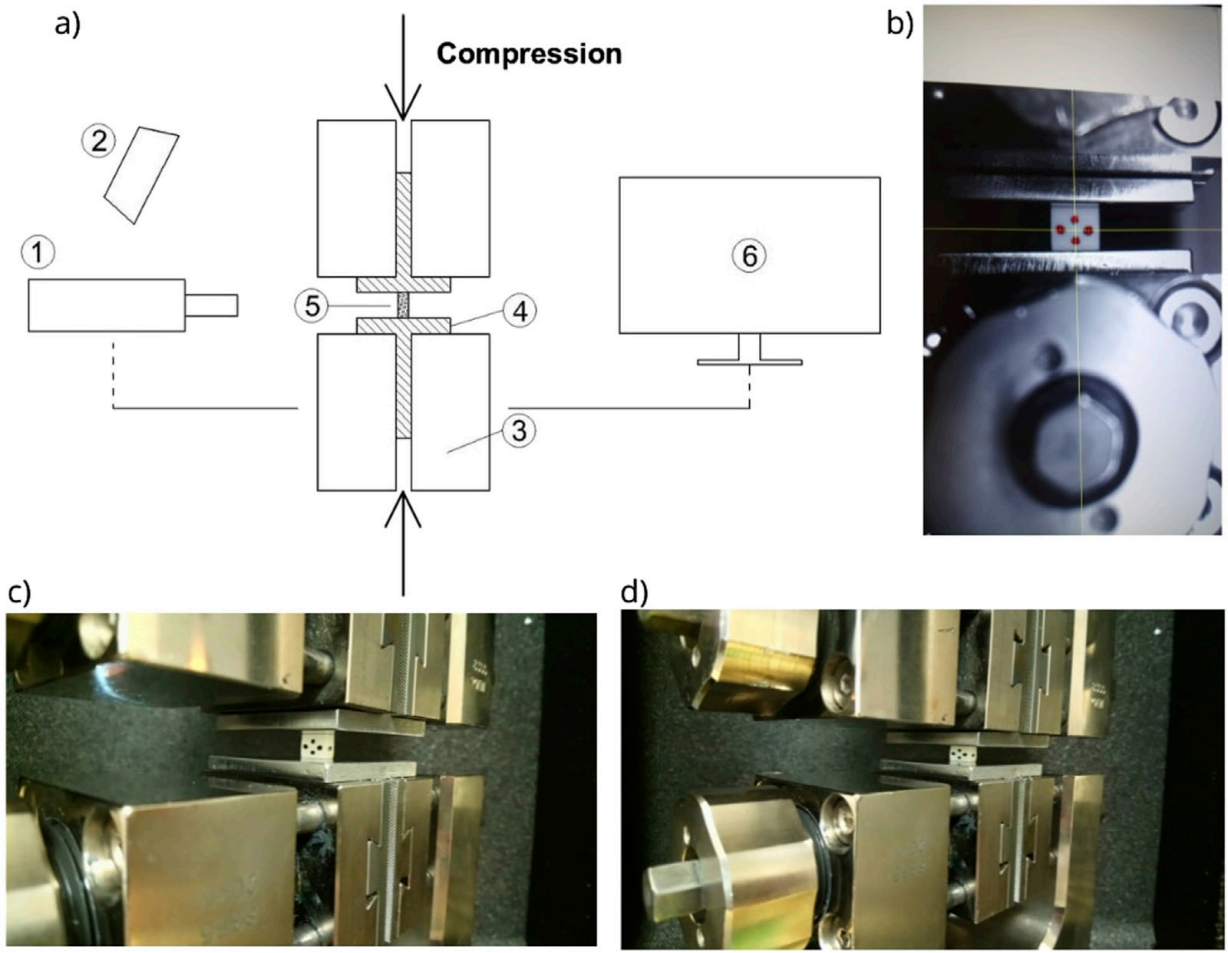
Compression tests: **(A)** schematic representation of the testing stand (1, video extensometer; 2, light; 3, machine testing grips; 4, flat inserts; 5, specimen; and 6, computer with TestXpert software), **(B)** view in the video extensometer with four markers on the specimen, **(C)** specimen before the test, and **(D)** specimen after the test.

A universal testing machine, Zwick/Roell Z0/20 (Zwick GmbH and Co. KG, Ulm, Germany), with a video extensometer (Zwick GmbH and Co. KG, Ulm, Germany) was used for the monotonic uniaxial loading experiments (Figure 3).

**FIGURE 4:**
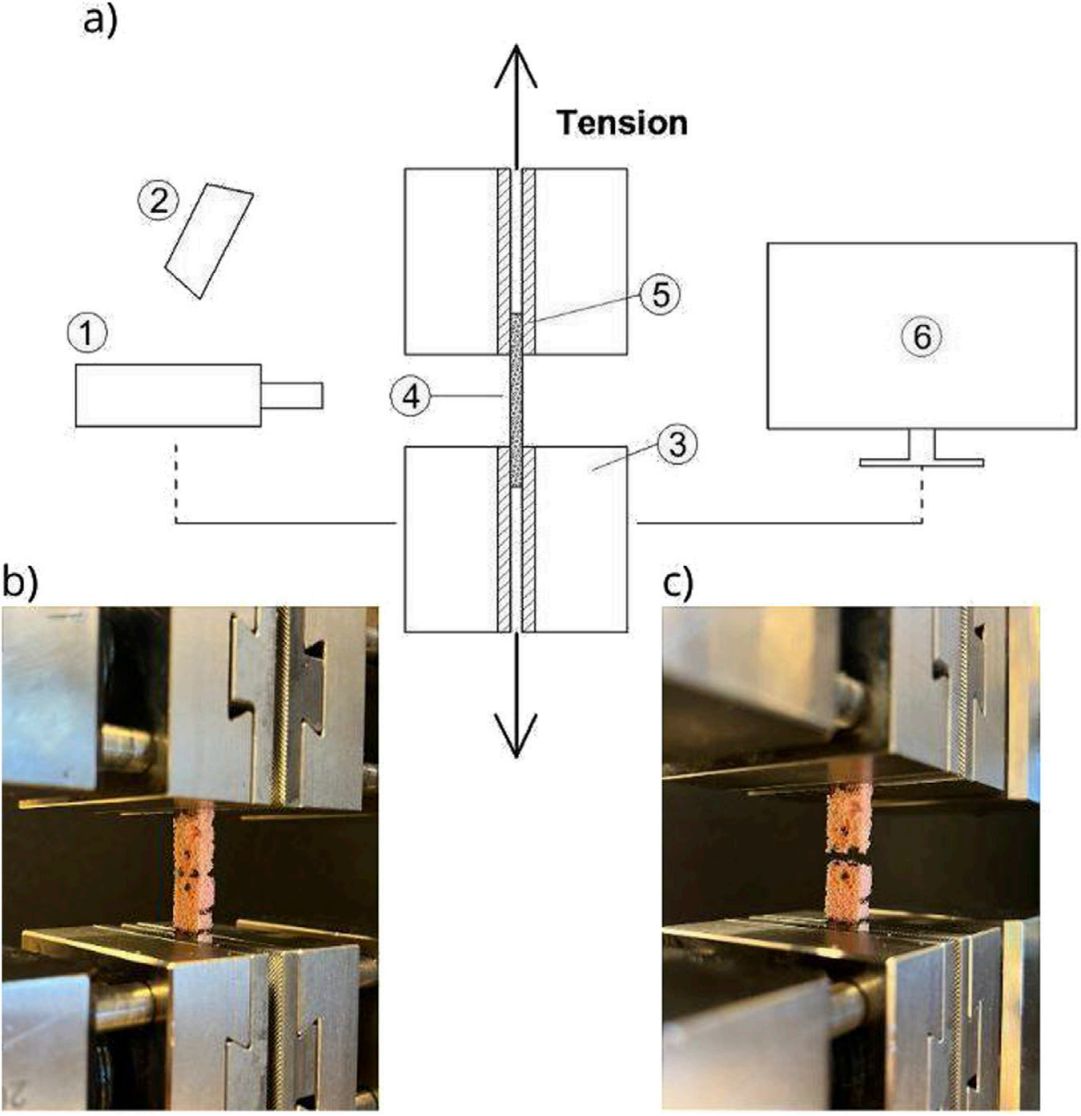
Tension tests: **(A)** schematic representation of the testing stand (1, video extensometer; 2, light; 3, machine testing grips; 4, specimen; 5, rugged inserts; and 6, computer with TestXpert software), **(B)** specimen before the test, and **(C)** specimen after the test. Parameter identification.

Special flat inserts were used for compression tests (Figure 3). For tensile tests, the specimens were clamped in dedicated Zwick/Roell rugged inserts that prevented any slippage during the test (Figure 4). The clamp distance was about 30 mm (three times greater than the specimen width of 10 mm) to guarantee the uniaxial uniform loading distribution in the central part of the specimen. Next, four markers were placed in the middle zone so the video extensometer could trace the sample deformation (Figure 3). The application of the video extensometer, which follows only the displacements of the markers put in the middle zone of the samples, minimizes the influence of possible friction between the specimen and clamps and gives more reliable results than measurements based on the displacement of the machine grips. The initial load was always assumed 2 N, and the test was load controlled with the rate of 1 mm/min according to the ISO 604 and ASTM D-695 standard recommendations (EN ISO 604, 2002) (ASTM, 2015). The same loading parameters were used for both specimens to allow direct comparison of compressive and tensile properties.

For compression loading, the test lasted to a strain of approximately 10%, while for tensile loading, the tests were carried out till the fracture of the specimen.

The different densities of the PU foams analyzed in the current study have different pore and cell sizes. Some samples were very fragile and broke during montage in the clamps or at the very beginning of the test. Only the specimens that underwent the full loading procedure and broke in the middle of the specimen (between markers) were taken for further analysis.

### 2.3 Parameter identification

Normal stress σ = F/A was calculated as force F obtained from the testing machine acting on the specimen cross-section area A and was calculated for each specimen separately. Longitudinal strain ε_L_ = ΔL_L_/L_L0_ was obtained based on the initial longitudinal distance L_L0_ and change between markers ΔL_L_ recorded in the loading direction between the markers that were tracked by the video extensometer. Transverse strain ε_T_ = ΔL_T_/L_T0_ was obtained based on the initial distance L_T0_ and the change between markers ΔL_T_ recorded in the direction transverse to the loading one. Stress–longitudinal strain σ/ε_L_ curves were built, and the linear part was approximated by the linear function. The slope of the approximation line was taken as the elastic modulus *E*. The yield stress σ_pl_ was calculated as the stress level corresponding to 0.2% of the plastic strain. For tensile tests, the rupture of the specimens often occurred before reaching the yield point; in such cases, the yield stress was not reported at all. For compressive tests, the maximum stress observed in the range of 0%–10% of strain was taken as the ultimate compressive strength *σ*_US_^C^ according to standard D 1621 (ASTM, 2023). For tensile tests, the rapture stress was taken as ultimate tensile strength σ_US_^T^. Next, the longitudinal strain –transverse strain ε_L_/ε_T_ curves were built, and the linear part was approximated by the linear function in the same range of longitudinal strains as for the identification of elastic modulus performed before. Linear elastic behavior of the tested material is assumed within this range. Finally, the slope of the approximation line was taken as Poisson’s ratio *ν*.

### 2.4 Statistical analysis

The influence of the loading direction on the compressive modulus, Poisson ratio, yield stress, and ultimate strength was assessed by the ANOVA (significance level *p*=< 0.001) for samples with normal distribution and by the ANOVA on ranks for samples without normal distribution. The pairwise multiple comparison procedure was performed using the Holm–Sidak method (normal distribution) or the Tukey test (non-normal distribution) to identify the difference between groups. The overall significance level was always *p* = 0.05.

Student’s t-test was used to compare the mechanical parameters obtained between tension and compression loading. The Student’s t-test was also used to compare parameters between particular models separately for tension and compression. The overall significance level for the Student’s t-test was always *p* = 0.001.

## 3 Results

**FIGURE 5:**
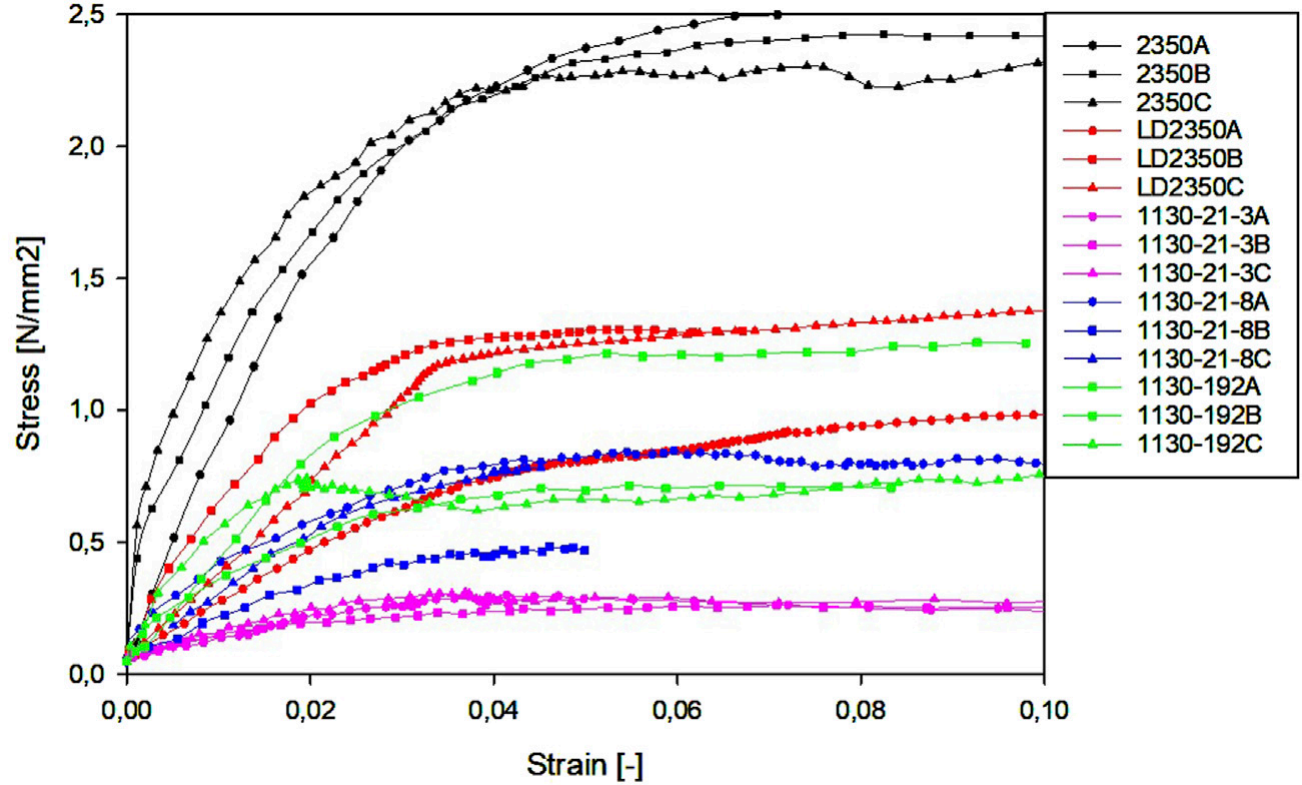
Representative stress–strain curves for compression tests.

**FIGURE 6:**
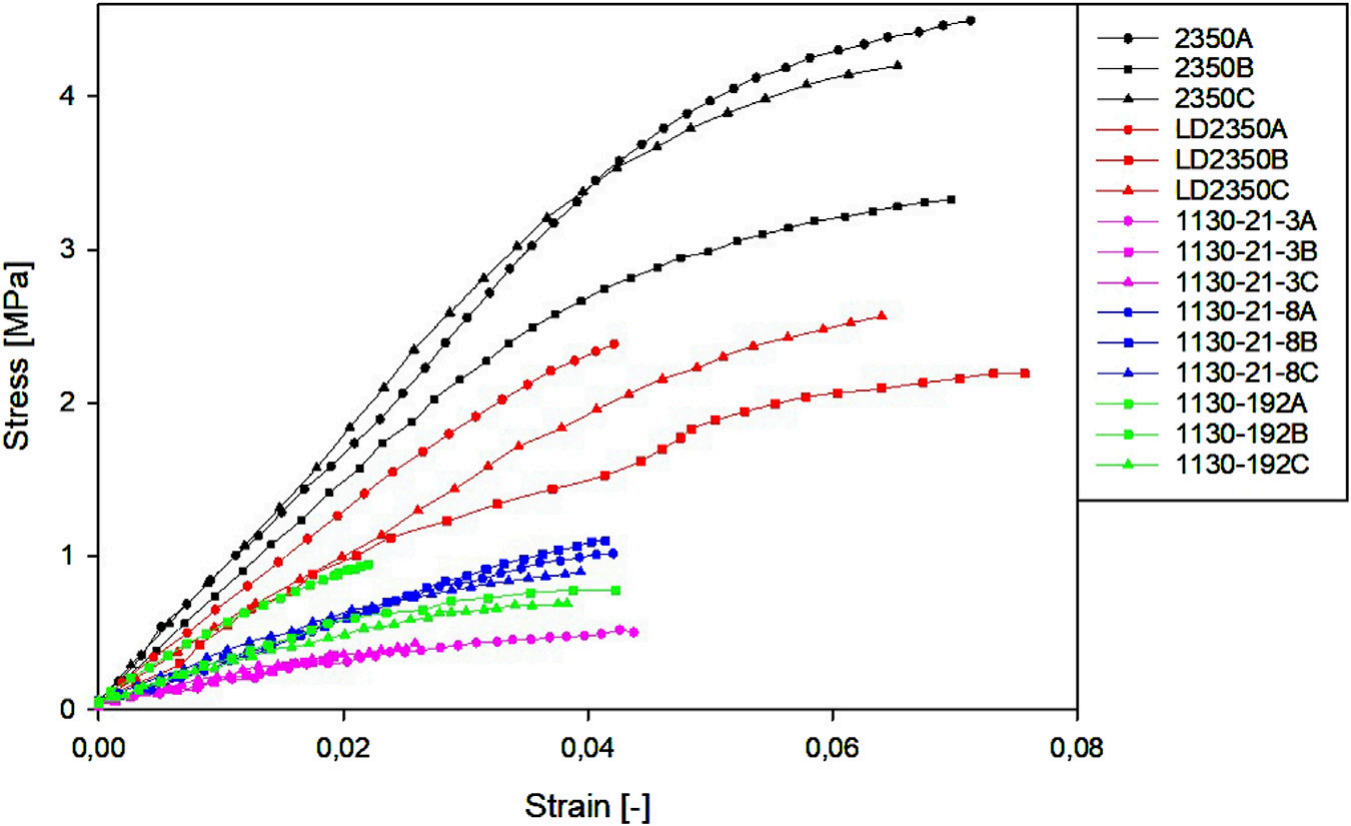
Representative stress–strain curves for tension tests.

Representative stress–strain graphs for the compression and tension tests are presented in Figures 5, 6, respectively.

The values of the obtained elastic modulus, Poisson ratios, yield stress, and ultimate strength for all the tested groups are collected in Table 2 and Table 3 for compression and tensile tests, respectively. The results in the separately tested groups A, B, and C followed the normal distribution (Shapiro–Wilk test, *p* < 0.05). The number of specimens N for tensile tests and the yield limit parameter is sometimes different than for the other parameters, as several times, the specimen fractured before reaching the yield limit. In such a case, the yield limit was missed, but the ultimate tensile strength was reported.

**TABLE 2:** Compressive material properties of tested PU foams.

Bone model	App. Density *ρ*_app_	Compressive elastic modulus *E* ^C^					Poisson’s ratio *ν* ^C^					Yield stress *σ*_pl_^C^					Ultimate compressive strength *σ*_US_^C^				
	[g/cm^3^]	Mean (SD), [MPa]			ANOVA	Post-hoc	Mean (SD), [MPa]			ANOVA	Post-hoc	Mean (SD), [MPa]			ANOVA	Post-hoc	Mean (SD), [MPa]			ANOVA	Post-hoc
		A	B	C	*p*-value	*p*-value	A	B	C	*p*-value	*p*-value	A	B	C	*p*-value	*p*-value	A	B	C	*p*-value	*p*-value
SYNBONE 2350	0.17 (0.02)	73.8 (7.0) *N = 8*	81.6 (8.2) N = 8	86.7 (21.8) N = 8	0.101	—	0.29 (0.03) N = 8	0.34 (0.04) N = 8	0.34 (0.05) N = 8	0.058	—	1.77 (0.19) N = 8	1.72 (0.18) N = 8	1.94 (0.28) N = 8	0.133	—	2.44 (0.15) N = 8	2.18 (0.18) N = 8	2.41 (0.26) N = 8	0.065	—
SYNBONE LD2350	0.13 (0.01)	18.7 (7.2) *N =* 8	46.8 (13.0) *N = 7*	31.0 (10.1) *N = 7*	<0.001	B vs. A (<0.001) B vs. C (0.019) C vs. A (0.031)	0.15 (0.06) *N =* 8	0.40 (0.05) *N = 7*	0.23 (0.03) *N = 7*	<0.001	B vs. A (<0.001) B vs. C (<0.001) C vs. A (0.008)	0.67 (0.17) *N =* 8	1.15 (0.17) *N = 7*	1.02 (0.19) *N = 7*	<0.001	B vs. A (<0.001) C vs. A (0.002)	0.86 (0.23) *N =* 8	1.36 (0.24) *N = 7*	1.38 (0.22) *N = 7*	<0.001	B vs. A (<0.001) C vs. A (<0.001)
Sawbones 1130-21-8	0.07 (0.01)	21.1 (7.7) N = 8	18.3 (5.9) N = 8	23.3 (4.7) N = 8	0.296	—	0.31 (0.08) N = 8	0.28 (0.03) N = 8	0.33 (0.05) N = 8	0.155	—	0.64 (0.12) N = 8	0.56 (0.14) N = 8	0.69 (0.04) N = 8	0.077	—	0.70 (0.13) N = 8	0.62 (0.16) N = 8	0.77 (0.03) N = 8	0.220	—
Sawbones 1130-21-3	0.05 (0.01)	8.2 (2.1) N = 8	8.7 (1.6) N = 8	9.5 (1.5) N = 8	0.342	—	0.30 (0.06) N = 8	0.30 (0.06) N = 8	0.30 (0.08) N = 8	0.985	—	0.27 (0.02) N = 8	0.24 (0.04) N = 8	0.29 (0.01) N = 8	0.044	B vs. C (0.044)	0.29 (0.01) N = 8	0.26 (0.03) N = 8	0.31 (0.01) N = 8	<0.001	B vs. C (<0.001)
Sawbones 1130-192	0.14 (0.01)	50.1 (19.4) N = 5	49.8 (34.3) N = 7	46.1 (34.5) N = 5	0.973	—	0.31 (0.13) N = 5	0.26 (0.04) N = 7	0.32 (0.20) N = 5	0.708	—	1.01 (0.29) N = 5	0.71 (0.43) N = 7	0.68 (0.21) N = 5	0.265	—	0.94 (0.46) N = 5	1.07 (0.57) N = 7	0.74 (0.18) N = 5	0.287	—

**TABLE 3:** Tensile material properties of tested PU foams.

Bone model	App. Density *ρ*_app_	Tensile elastic modulus *E* ^T^					Poisson’s ratio *ν* ^T^					Yield stress *σ*_pl_^T^					Ultimate tensile strength *σ*_US_^T^				
	[g/cm^3^]	Mean (SD), [MPa]			ANOVA	Post-hoc	Mean (SD), [MPa]			ANOVA	Post-hoc	Mean (SD), [MPa]			ANOVA	Post-hoc	Mean (SD), [MPa]			ANOVA	Post-hoc
		A	B	C	*p*-value	*p*-value	A	B	C	*p*-value	*p*-value	A	B	C	*p*-value	*p*-value	A	B	C	*p*-value	*p*-value
SYNBONE 2350	0.18 (0.01)	87.2 (12.2) *N =* 4	70.6 (5.5) N = 4	89.4 (27.3) N = 4	0.301	—	0.31 (0.04) N = 4	0.31 (0.03) N = 4	0.32 (0.05) N = 4	0.817	—	3.34 (0.74) N = 4	3.16 (0.47) N = 4	3.27 (0.16) N = 4	0.887	—	3.79 (0.86) N = 4	3.63 (0.26) N = 4	4.04 (0.29) N = 4	0.578	—
SYNBONE LD2350	0.14 (0.01)	70.5 (26.9) *N* = 6	41.5 (13.2) *N* = 4	49.7 (8.4) *N* = 4	0.100	—	0.31 (0.04) N = 6	0.28 (0.15) N = 4	0.35 (0.06) N = 4	0.588	—	2.14 (0.75) N = 5	1.32 (0.19) N = 4	1.60 (0.85) N = 4	0.234	—	2.53 (0.51) *N = 6*	2.08 (0.64) *N = 4*	2.18 (0.52) *N = 4*	0.359	—
Sawbones 1130-21-8	0.10 (0.01)	27.1 (6.3) *N* = 5	26.9 (2.0) *N* = 5	29.0 (6.1) *N* = 4	0.804	—	0.34 (0.12) N = 5	0.35 (0.14) N = 5	0.35 (0.09) N = 4	0.985	—	0.86 (0.09) N = 3	0.89 (0.15) N = 2	0.81 (0.18) N = 2	0.842	—	1.00 (0.04) N = 5	0.94 (0.13) N = 5	0.98 (0.09) N = 4	0.841	—
Sawbones 1130-21-3	0.05 (0.01)	13.3 (3.7) *N* = 8	14.2 (3.6) *N* = 7	14.8 (4.6) *N* = 8	0.768	—	0.34 (0.15) N = 8	0.41 (0.09) N = 7	0.38 (0.06) N = 8	0.602	—	0.40 (0.05) N = 5	0.37 (0.02) N = 3	0.42 (0.08) N = 2	0.571	—	0.42 (0.08) N = 8	0.41 (0.04) N = 7	0.41 (0.07) N = 8	0.906	—
Sawbones 1130-192	0.15 (0.02)	50.2 (33.7) *N* = 8	32.8 (26.4) *N* = 7	22.4 (8.1) *N* = 8	0.107	—	0.23 (0.14) N = 8	0.21 (0.14) N = 7	0.28 (0.09) N = 8	0.716	—	0.65 (0.40) N = 5	0.61 (0.30) N = 4	0.70 (0.18) N = 5	0.908	—	1.02 (0.17) N = 8	0.90 (0.17) N = 7	0.71 (0.21) N = 8	0.010	C vs. A (0.01)

**FIGURE 7:**
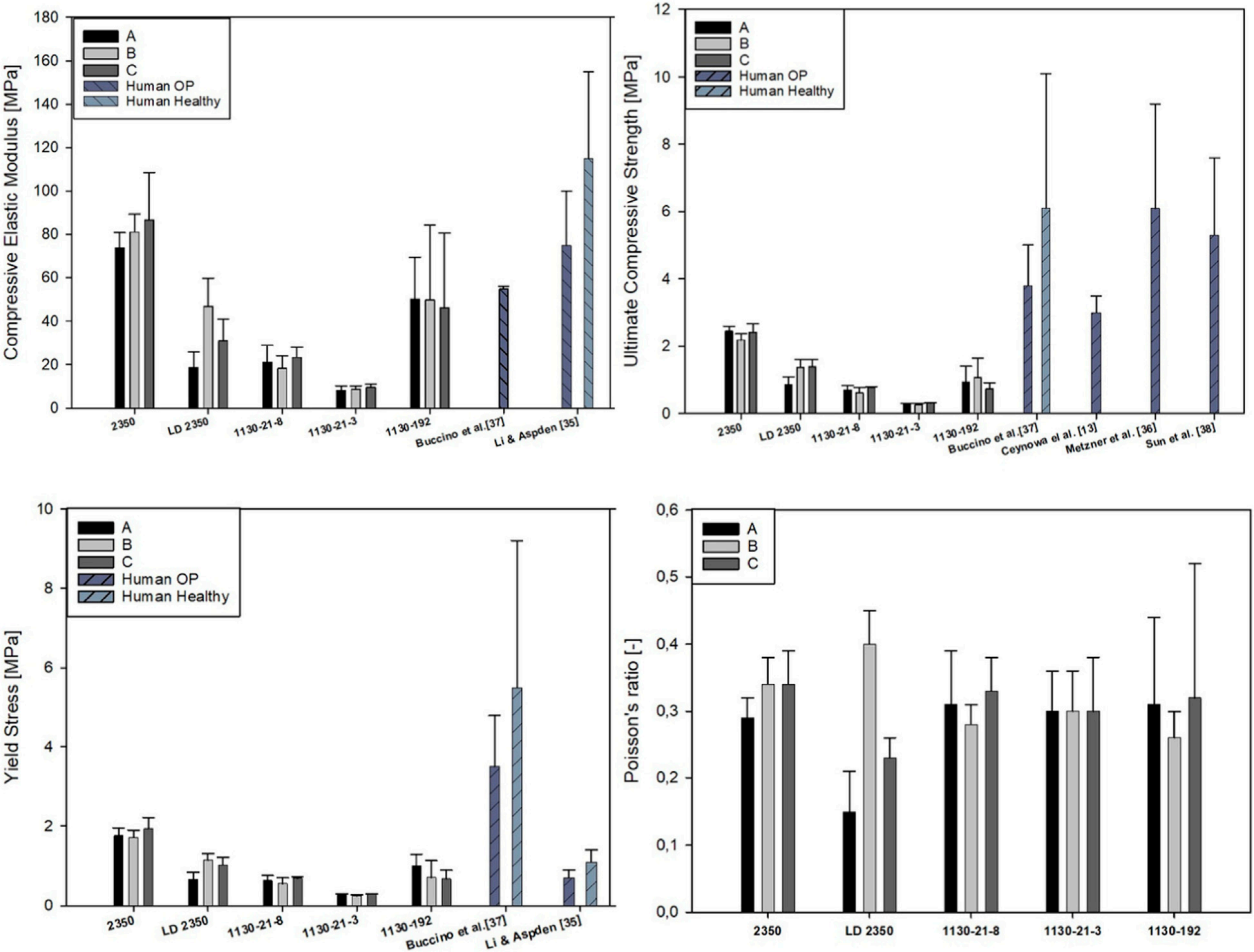
Compressive mechanical properties for tested PU foams of different bone analogs compared with results from the literature.

**FIGURE 8:**
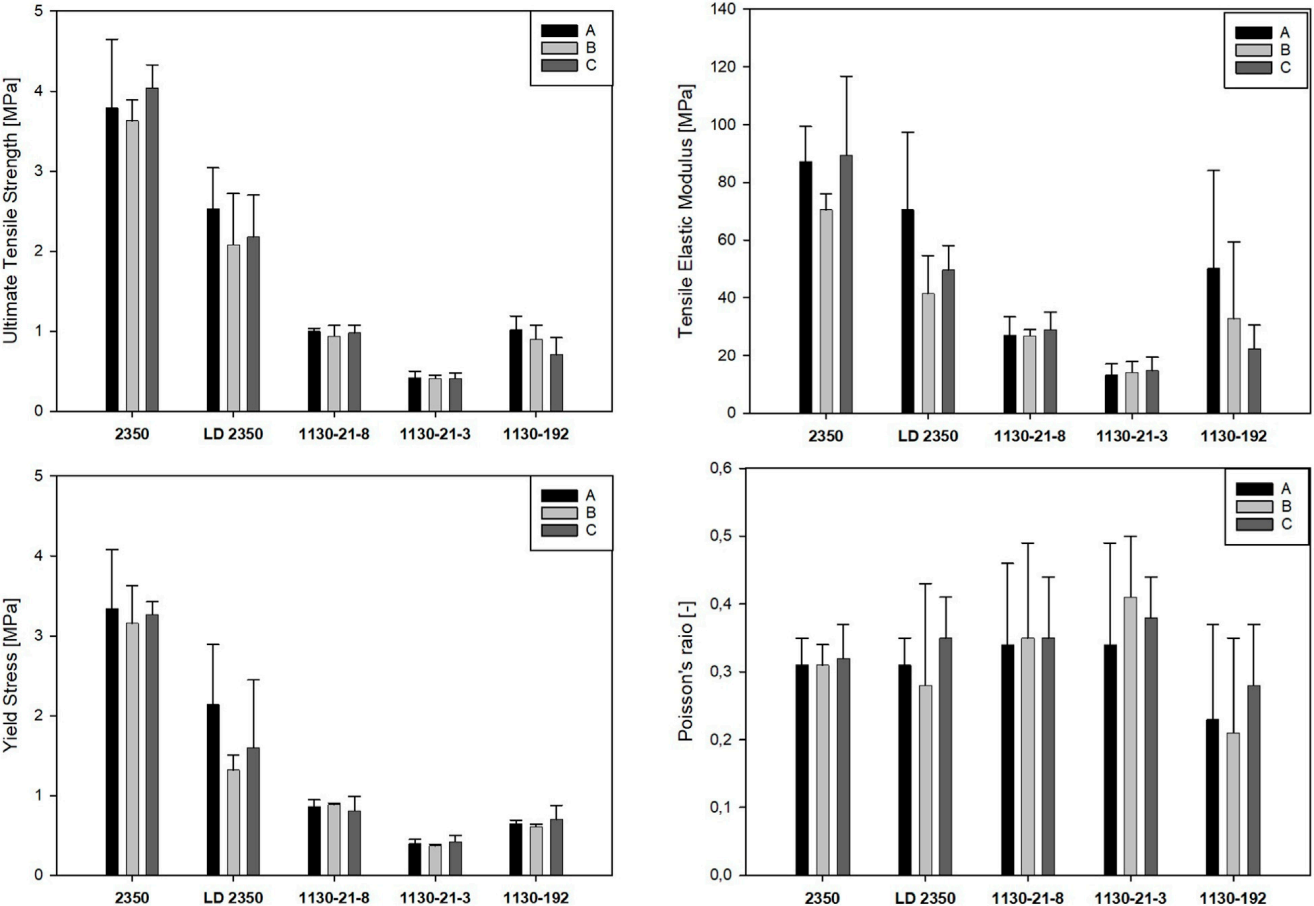
Tensile mechanical properties of tested PU foams for different bone analogs.

In Figures 7, 8, the bar plots for the obtained mechanical parameters of the PU foams mimicking the trabecular tissue in the femoral head are presented, distinguished by product name and direction of specimen cutting, for the compression and tension tests, respectively. The bar presents the mean value, and the whiskers represent the standard deviation.

There were significant differences for all the compressive properties between groups A, B, and C (ANOVA test *p* < 0.001) only for the SYNBONE LD2350 bone analog. However, for the same bone model and tensile tests, there were no significant differences between directions A, B, and C. The remaining synthetic femurs exhibited isotropic character as there were no statistically significant differences between the elastic modulus, Poisson ratios, yield stress, and ultimate strength among directions A, B, and C under both compressive and tensile loading. Only some differences in ultimate strength were observed for tension in the 1130-192 model (directions C vs. B) and compression in the 1130-21-3 model (directions C vs. B). Being non-isotropic, the PU foam used in the SYNBONE LD2350 model best mirrors the non-homogenous and non-isotropic character of the osteoporotic type of human trabecular tissue under compression.

The comparison between compression and tension loading for the same bone analog revealed that the Sawbones 1130-192 model exhibits the same properties regardless of loading direction (*t*-test, *p* > 0.001). Significant differences between compression and tension regarding yield limit and ultimate strength were observed for Sawbones 1130-21-3, Sawbones 1130-21-8, and SYNBONE 2350 (*t*-test, *p* < 0.001). Similarly, the SYNBONE LD2350 direction A model gave different results in terms of elastic modulus, yield limit, and ultimate strength. The remaining parameters were unchanged and were not related to the loading character (*t*-test, *p* > 0.001).

Applying the paired Student’s t-test between all the models for all the parameters revealed that the differences regarding elastic modulus, yield limit, and ultimate strength are statistically significant, both for tension and compression loading. Only differences regarding the Poisson ratios were not statistically significant between analyzed models.

## 4 Discussion

Five different types of synthetic bones from two leading bone analog producers were investigated in the current study. SYNBONE does not reveal the detailed mechanical properties of their products; they only provide a general statement that the cortical layer is of low density (LD2350) and the cancellous layer is soft (2350, LD2350), which probably corresponds to the cellular type of the foam. For the Sawbones products, the information about foam density mimicking the cancellous tissue in the proximal femur end is reported as 3 PCF and 7 PCF for the 1130-21-3 and 1130-21-8 products, respectively. In the distal end, the foam is called “standard cancellous” without further details. The 1130-192 bone analog lacks information on foam density or type. No further details are published. It is known that solid rigid PU foams of grades 5, 10, 12, 15, 20, 25, 30, 35, 40, and 50 produced by Sawbones conform to ASTM F-1839-08 “Standard Specification for Rigid Polyurethane Foam for Use as a Standard Material for Testing Orthopaedic Devices” (ASTM, 2014), but detailed information on which grade is used for a particular model is often missing. Sometimes, the producer only reports information about the density of the foams used. For the production of bone surrogates, SYNBONE uses foams of density 0.08–0.80 g/cm^3^ (ISO1183) with corresponding compressive strength 0.7–55 MPa (ISO 604, ASTM D-695).

These products are mainly dedicated to training medical staff; therefore, the principal aim of the producer is to obtain a tactile experience equivalent to human natural tissue during hands-on training for techniques such as cutting, screwing, plating, or nailing of the implants. However, many biomechanical studies have been conducted on these models, obtaining excellent and convergent results of medical importance (Ceynowa et al., 2020a; Ceynowa et al., 2021b). Therefore, research on the mechanical properties of the bone analogs and their similarities and differences with natural human tissue is of key importance to ensure that the experimental testing of the implant accurately reflects the conditions of real human bone.

The presented research aimed at investigating the mechanical properties of PU foams mimicking trabecular tissue in the commercially available composite femurs of different producers.

**TABLE 4:** ASTM F-1839-97 requirements for rigid PU foams.

Grade	Designation and density		Requirements for compressive strength		Requirements for compressive modulus	
	Minimum density [kg/m^3^]	Maximum density [kg/m^3^]	Minimum compressive strength [MPa]	Maximum compressive strength [MPa]	Minimum compressive modulus [MPa]	Maximum compressive modulus [MPa]
5	72.1	88.1	0.4495	0.78	12.30	20.35
10	144.0	176.0	1.7450	2.82	45.75	71.70
12	173.0	211.5	2.4850	3.97	64.50	100.50

The direct conclusions are as follows:

- the PU foam in the SYNBONE LD2350 femur model can be classified between grade 5 and grade 10 of the ASTM F-1839-08 (Table 4) standard considering density, compressive modulus, and compressive strength.
- the PU foam in the SYNBONE 2350 femur model can be classified closer to grade 10 of the ASTM F-1839-08 standard, taking into account density and compressive strength into account, and to grade 12 when taking the compressive modulus into account.
- the PU foam in the Sawbones 1130-21-3 femur model is much lower than grade 5 of the ASTM F-1839-08 standard, taking compressive strength and compressive modulus into account, but meets the producer specification of 3 PCF (about 0.048 g/cm^3^).
- the PU foam in the Sawbones 1130-21-8 femur model has a density a bit less than reported by the supplier of 7 PCF (about 0.112 g/cm^3^) and can be classified close to grade 5 of the ASTM F-1839-08 standard, taking into account density, compressive strength, and compressive modulus.
- the PU foam in the Sawbones 1130-192 femur model can be classified close to grade 10 of the ASTM F-1839-08 standard, taking into account density and compressive modulus, and between grade 5 and grade 12 when taking the compressive strength into account.

In Table 5, the mechanical parameters identified in the current study are compared with the values found by the other researchers for the natural human healthy and osteoporotic trabecular tissue, as well as with some other PU foams designed to mimic trabecular osteoporotic tissue. Only studies regarding direct mechanical testing on macroscale specimens were included.

**TABLE 5:** Mechanical properties of the PU foams mimicking trabecular tissue and human osteoporotic and healthy trabecular tissue under mechanical compression loading.

References	Bone type	Comments	Density	Compressive elastic modulus	Poisson’s ratio	Yield stress	Ultimate compressive strength
			[g/cm^3^]	[MPa]	[-]	[MPa]	[MPa]
Current study							
SYNBONE 2350	artificial	cuboid 10 mm × 10 mm × 4 mm	0.17 ± 0.02	70.7 ± 12.3	0.32 ± 0.40	1.81 ± 0.22	2.34 ± 0.20
SYNBONE LD2350	artificial		0.13 ± 0.01	32.2 ± 10.1	0.26 ± 0.05	0.95 ± 0.18	1.2 ± 0.23
Sawbones 1130-21-3	artificial		0.05 ± 0.01	8.8 ± 1.73	0.3 ± 0.07	0.28 ± 0.02	0.29 ± 0.02
Sawbones 1130-21-8	artificial		0.07 ± 0.01	20.9 ± 6.1	0.31 ± 0.05	0.63 ± 0.23	0.70 ± 0.11
Sawbones 1130-192	artificial		0.14 ± 0.01	48.7 ± 29.4	0.30 ± 0.12	0.8 ± 0.31	0.92 ± 0.40
Marter et al. (2019)	artificial cellular PU foams Sawbones code 1522	cuboid 51 mm × 51 mm × 40 mm, yield stress taken for maximum force two directions of compression (x), (y)	0.12 ± 0.01	55.5 ± 8.3^(x)^ 38.8 ± 3.0^(y)^	0.41 ± 0.08^(x)^ 0.33 ± 0.02^(y)^	N/A	N/A
			0.20 ± 0.02	160.0 ± 6.4^(x)^ 120.0 ± 3.6^(y)^	0.39 ± 0.02^(x)^ 0.31 ± 0.01^(y)^	N/A	N/A
			0.24 ± 0.02	212.0 ± 3.8^(x)^ 164.0 ± 2.5^(y)^	0.34 ± 0.02^(x)^ 0.31 ± 0.01^(y)^	N/A	N/A
Patel et al. (2008)	Artificial PU foams from Sawbones	Cylinder Φ9 mm × 3. mm ^(1)^ 9 mm × 7.7 mm ^(2)^	0.09	0.3 ± 0.2^(1)^ 0.7 ± 0.2^(2)^	N/A	0.02 ± 0.01^(1)^ 0.04 ± 0.02^(2)^	N/A
			0.16	19 ± 3^(1)^ 41 ± 3^(2)^	N/A	1.0 ± 0.01^(1)^ 1.1 ± 0.1^(2)^	N/A
			0.32	66 ± 13^(1)^ 145 ± 6^(2)^	N/A	3.6 ± 0.5^(1)^ 3.3 ± 0.9^(2)^	N/A
Calvert et al. (2010)	Artificial PU foams from General Plastics, Tacoma, WA	Cylinder Φ7.55 mm × 15 mm	0.24	134 ± 9	N/A	N/A	4.8 ± 0.1
			0.30	216 ± 17	N/A	N/A	8.5 ± 0.3
			0.32	206 ± 12	N/A	N/A	8.2 ± 0.4
			0.40	356 ± 25	N/A	N/A	13.5 ± 0.2
			0.64	752 ± 43	N/A	N/A	24.6 ± 0.3
Palissery et al. (2004)	Artificial PU foams from HEREX C.70.55	Cylinder Φ15 mm × 17 mm cyclic loading	N/A	39.6 ± 1.2	N/A	N/A	0.64 + 0.02
Li and Aspden (1997)	Human osteoporotic	Cylinder Φ9 mm × 7.7 mm	0.18–0.39	75 ± 25	N/A	0.7 ± 0.2	N/A
Metzner et al. (2021)	Human osteoporotic	Cylinder Φ8 mm × 16 mm	0.3–0.85	647 ± 300	N/A	N/A	6.1 ± 3.1
Buccino et al. (2021)	Human osteoporotic	Cuboid 4 mm × 4 mm × 14 mm	N/A	55 ± 1	N/A	3.5 ± 1.3	3.8 ± 1.2
Sun et al. (2008)	Human osteoporotic	Cuboid 10 mm × 10 mm × 10 mm	1.06 ± 0.13 (BMD))	339 ± 178	N/A	N/A	5.3 ± 2.3
Ceynowa et al. (2020b)	Human osteoporotic	Cuboid 12 mm × 12 mm × 27	1.00 ± 0.06	N/A	N/A	N/A	3.0 ± 0.5
Li and Aspden (1997)	Human healthy	Cylinder φ9 mm × 7.7 mm	0.21–0.42	115 ± 40	N/A	1.1 ± 0.3	N/A
Metzner et al. (2021)	Human healthy	Cylinder φ8 mm × 16 mm	0.67–1.16	604 ± 200	N/A	N/A	14.1 ± 7.3
Martens et al. (1983)	Human healthy	Cylinder φ8 mm × 8 mm The direction of compression: (x), (y), (z)	N/A	900 ± 710^(x)^ 811 ± 604^(y)^ 404 ± 66^(z)^	N/A	N/A	9.3 ± 4.5^(x)^ 10.2 ± 3.3^(y)^ 4.9 ± 1.3^(z)^
Buccino et al. (2021)	Human healthy	cuboid 4 mm × 4 mm × 14 mm	N/A	N/A	N/A	5.5 ± 3.7	6.1 ± 4.0

The range of osteoporotic trabecular tissue properties for human trabecular tissue in the femoral head is quite broad across other studies. It depends on the stage of osteoporosis that affects the mineral density of the bone and, therefore, directly impacts bone strength.

Li and Aspden (1997) reported detailed values of normal (NOR), osteoarthritis (OA), and osteoporotic (OP) types of human cancellous bone retrieved from the femoral head. However, it is difficult to draw direct conclusions between that study and the current research, as the values from the first study were not normally distributed; thus, only median and ranges were reported. However, the results obtained in the current study are within the ranges of the elastic modulus (OP 50–410 MPa, NOR 40–460 MPa) and yield strength (OP 0.6–5.8 MPa, NOR 0.4–9.0 MPa) reported by Li and Aspden (1997). On the other hand, the values reported by Li and Aspden (1997) are very similar for normal and osteoporotic bone, thus making a clear distinction between these bones difficult. It should be noted that the yield limit was identified as in the present study, but the yield strength (Li and Aspden, 1997) was the stress corresponding to the 3% drop of the maximum stiffness. The apparent density of the foams in the current study was in the range of 0.049–0.176 g/cm^3^; that is much lower than for the human tissue reported to be 0.47 g/cm^3^ and 0.38 g/cm^3^ for normal and OP bone (Li and Aspden, 1997), respectively.

Sun et al. (2008) also compared the properties of trabecular femoral bone obtained from patients with OA and OP. They cut the specimens from the femoral head in the vertical direction of the coronal plane, which corresponds to the B direction of the present study. The mean values of the compressive elastic modulus were 829 MPa (SD 160) and 363 MPa (SD 131) for the OA and OP patients, respectively. These values are much higher than the ones obtained in the current study.

Buccino et al. (2021) performed very detailed research on the trabecular tissue characterization of OA and OP patients, taking into account the localization of the tissue. First, the femoral heads were cut in the coronal plane as in the current study. Then, relatively small specimens (4 mm × 4 mm × 16 mm) were extracted according to the stress trajectories in the femoral head. These specimens underwent micro-compression tests and post data processing according to the innovative procedure proposed by the authors. One of the main conclusions stated that osteoporotic bones had a non-uniform distribution of Young’s modulus values across the femoral head.

Ceynowa et al. (2020c) tested osteoporotic bone retrieved from human femoral heads along the axis of lag screw positioning. The influence of drilling the trabecular tissue with surgical guide wire on the overall strength of the bone blocks was investigated. The specimens were cut from the femoral head. The results obtained for the undrilled samples were as follows: mean density 1 g/cm^3^ and ultimate compressive strength approximately 3 MPa. In the current research, the obtained values of densities for all the tested surrogate tissue were much lower (range 0.05–0.18 g/cm^3^), but the compressive strength of the femur model SYNBONE 2350 was quite similar (range 2.18–2.44 MPa).

Palissery et al. (2004) found that the tensile properties were greater than the compressive properties for the HEREX PU foams of different grades tested under cyclic loading. In the present study, there were no differences between the tensile and compressive elastic moduli (apart from the 1130-21-3 model) and for the Poisson ratios for all models, but the yield limit and ultimate strength for all models except for the 1130-192 model were greater in tension than compression. However, there is still a debate about whether the compressive and tensile properties of the human trabecular tissue are equal or not (Palissery et al., 2004). This hypothesis requires further comprehensive research.

Patel et al. (2008) suggested that Sawbones PU foam of 0.16 g/cm^3^ density is appropriate to mimic the cancellous OP bone for fracture stress analysis but not for energy dissipation. This foam has an elastic compression modulus of 19 MPa and 41 MPa and yield strength of 1.0 MPa and 1.1 MPa, depending on the length of the sample. In the current study, similar compressive properties were found for the Sawbones 1130-192 bone model density of about 0.14 g/cm^3^, a mean elastic modulus of 48.8 MPa, and an average yield limit of 0.79 MPa. The SYNBONE LD2350 bone model, characterized by anisotropy properties, had a density of 0.13 g/cm^3^, an elastic compressive modulus in the range of 18.5–46.8 MPa, and a yield limit in the range of 0.66 MPa–1.15 MPa. To sum up, these two bone models are suggested to simulate the OP behavior of the cancellous bone similarly to that suggested by Patel et al. (2008). However, studies regarding OP bone are rare, and the differences between individual humans can span a broad range of values; thus, the conclusion must be taken with utmost care. Moreover, Patel et al. (2008) proved that sample dimensions had a great impact on the obtained results, which could also affect the comparisons made here.

Calvert et al. (2010) tested a wide range of grades of rigid PU foams manufactured by General Plastics (GP), Tacoma, WA. The same monotonic compressive tests were performed on block specimens (50.8 mm × 50.8 mm × 25.4 mm) according to ASTM F1839 standard as well as on smaller cylinder samples of 7 mm diameter × 15 mm height, as analog for specimens used for tests on natural bone tissue. Steorology was used for cell size and pore arrangements analysis, and, additionally, the hardness of the foams was measured. The cyclic tests were only performed on cylindrical specimens. The elastic modulus and compressive strength values increased with increasing density, and all the results met the ASTM F1839-08 standard ranges for particular grades. Cyclic tests indicated no strengthening between cycles; however, only ten cycles were performed. The differences between results obtained from larger and smaller specimens were not statistically important. The grades and densities of PU foams tested by Calvert et al. (2010) are much higher than those investigated in the current study; thus, a direct comparison is impossible.

Most of the tests on foam materials are performed parallel to the foaming direction, which is the standard orientation used for bone tissue analog manufacturing (Calvert et al., 2010). Marter et al. (2019) tested Sawbones PU foams under compressive loading with an optical extensometer to identify Young’s moduli and Poisson ratios of different types (solid, cellular, and open cell) and densities of foams. The samples were cut out of foam blocks in two directions, parallel and perpendicular to the foaming direction. The material was tested in one plane upon the assumption of transverse isotropy. The Young’s modulus values and Poisson ratios for cellular foams are higher when the foams are compressed parallel to the foaming direction than in the perpendicular direction. The open cell foams exhibit a reverse relationship, where the Young’s modulus values were lower for the parallel direction than for the perpendicular one, but the Poisson ratios were higher for the parallel direction than for the perpendicular. The solid foams showed no differences between loading directions for both moduli and thus were significantly isotropic across the different foam grades.

Only the results of the cellular foam type of 0.115 g/cm^3^ density (Marter et al., 2019) may be directly compared with the current results. Marter et al. (2019) determined Young’s modulus values of 55.5 MPa and 38.8 MPa and Poisson ratios of 0.41 and 0.33 for foaming and transverse loading directions, respectively, which are in accordance with our results on the Sawbones 1130-192 model, which had a density of 0.141 g/cm^3^ and a mean Young’s modulus of 48.8 MPa and a Poisson ratio of 0.29, both calculated as the mean values from the A, B, and C directions, as the ANOVA (*p* > 0.05) showed no differences between loading direction.

It could be assumed that the foaming direction is parallel to the shaft of the femur and to the direction C in the femoral head in the current study, but without any confirmation by the producer, this assumption cannot be taken for granted. It is clear that the manufacturing procedure regarding foaming has a great impact on the final properties of the material and its anisotropy/isotropy level, which is of key importance when these materials are used for clinical analysis of implants and orthopedic procedures and may affect the results regarding bone–implant interface and fixation stability. Sawbones developed a fourth-generation model of the human bones made of short fiber-filled epoxy for the cortical layer and PU foam for trabecular tissue that was thoroughly tested for application of orthopedics devices and implants and seems to be the best current model of the natural human bones (Zdero et al., 2023; Gluek et al., 2020; Aziz et al., 2014). However, the models made of PU foams of different densities and mechanical properties can be a much less expensive alternative (even ten times) for performing biomechanical experiments and giving promising results. The fact that these models are available in a wide range of geometries and with pre-defined fracture types should be taken as a great advantage, and thus, research on these models should continue.

The present work provides a detailed database of the mechanical properties of polyurethane foams mimicking the trabecular tissue in five synthetic bone models of the human femur from two different producers. These models can be further used for experimental research on orthopedic solutions. They can also serve for validation and calibration purposes of the numerical simulations of implants tested in the laboratory with these particular models.

## Data Availability

The original contributions presented in the study are included in the article/supplementary material; further inquiries can be directed to the corresponding author.
